# Effects of Bladder Cancer on UK Healthcare Costs and Patient Health-Related Quality of Life: Evidence From the BOXIT Trial

**DOI:** 10.1016/j.clgc.2019.12.004

**Published:** 2020-08

**Authors:** Edward Cox, Pedro Saramago, John Kelly, Nuria Porta, Emma Hall, Wei Shen Tan, Mark Sculpher, Marta Soares

**Affiliations:** 1 Centre for Health Economics, University of York, York, United Kingdom; 2 Division of Surgery and Interventional Science, University College London, London, United Kingdom; 3 Department of Urology, University College London Hospital, London, United Kingdom; 4 Clinical Trials and Statistics Unit, The Institute of Cancer Research, London, United Kingdom; 5 Department of Urology, Imperial College Healthcare, London, United Kingdom

**Keywords:** Cost, HRQoL, NMIBC, RCT, QALY

## Abstract

**Background:**

Limited evidence exists regarding the cost and health-related quality of life (HRQoL) effects of non–muscle-invasive bladder cancer (NMIBC) recurrence and progression to muscle-invasive bladder cancer (MIBC). We examined these effects using evidence from a recent randomized control trial.

**Material and Methods:**

The costs and HRQoL associated with bladder cancer were assessed using data from the BOXIT trial (bladder COX-2 inhibition trial; n = 472). The cost and HRQoL effects from clinical events were estimated using generalized estimating equations. The costs were derived from the recorded resource usage and UK unit costs. HRQoL was assessed using the EQ-5D-3L and reported UK preference tariffs. The events were categorized using the TMN classification.

**Results:**

Cases of grade 3 recurrence and progression were associated with statistically significant HRQoL decrements (−0.08; 95% confidence interval [CI], −0.13 to −0.03; and −0.10; 95% CI, −0.17 to −0.03, respectively). The 3-year average cost per NMIBC patient was estimated at £8735 (95% CI, 8325-9145). Cases of grade 1, 2, and 3 recurrence were associated with annual cost effects of £1218 (95% CI, 403-2033), £1677 (95% CI, 920-2433), and £3957 (95% CI, 2332-5583), respectively. Progression to MIBC was associated with an average increase in costs of £5407 (95% CI, 2663-8152).

**Conclusion:**

Evidence from the BOXIT trial suggests that patients with NMIBC will both experience decrements in HRQoL and incur significant costs, especially in the event of a grade 3 recurrence or a progression to MIBC.

## Micro-Abstract

Limited evidence exists regarding the costs and health-related quality of life (HRQoL) effects of bladder cancer. Our study derived both the mean and marginal UK HRQoL and cost effects across multiple grades and stages of bladder cancer using data from the BOXIT (bladder COX-2 inhibition trial; n = 472). We found that patients with bladder cancer will experience decrements in HRQoL, which impose significant costs in the event of disease recurrence or progression that increase with the abnormality and invasiveness of the lesion. The results of the present study will help to lay the foundation for future burden of disease studies and cost-effectiveness analyses.

## Introduction

Bladder cancer is the 9th most common cancer and ranks 13th in terms of cancer-associated mortality worldwide.^1^ In the United Kingdom, bladder cancer accounts for 3% of all new cancer cases, with an estimated 10,171 new cases diagnosed in 2015.^2^ Clinically, the lesions will be stratified using the TMN classification, with non–muscle-invasive bladder cancer (NMIBC) classified as stage Tis, Ta, and T1 and muscle-invasive bladder cancer (MIBC) as stage T2, T3, and T4. This distinction is important because the involvement of cancer invading the muscle carries a significantly worse prognosis and requires radical cystectomy, radical chemotherapy, or radical radiotherapy, with or without neoadjuvant chemotherapy. NMIBC has had more favorable survival rates but recurs frequently and has been associated with repeated outpatient visits, cytologic and cystoscopic monitoring, and adjuvant intravesical treatment regimens after transurethral resection.

In the European Union, it has been estimated that the treatment of bladder cancer costs €4.9 billion, representing 5% of the total healthcare cancer costs.^3^ In the United States, bladder cancer has been the most costly cancer to treat on a per patient basis.^4^^,^^5^ Having estimates of the cost and health-related quality of life (HRQoL) effects of clinical events related to bladder cancer is important as a means of understanding its burden, informing resource allocation decisions, and aiding further research. However, current evidence on such effects has been limited in several ways. First, the distinction between NMIBC recurrence and progression to MIBC has often been overlooked.^5^, ^6^, ^7^, ^8^ Second, HRQoL studies have predominantly focused on treatment-specific effects^6^, ^7^, ^8^, ^9^ and have not sought to understand the HRQoL effects of specific clinical events such as recurrence and progression. Third, systematic reviews have repeatedly criticized the internal validity of HRQoL analyses, commonly citing the use of retrospective or cross-sectional designs, nonvalidated instruments, short time horizons, and failure to adjust for confounders.^7^, ^8^, ^9^, ^10^, ^11^ Finally, a paucity of UK-specific cost analyses have been reported.

The present study aimed to estimate the expected costs and HRQoL of patients with a diagnosis of NMIBC and evaluate the effects associated with NMIBC recurrence and progression to MIBC. Our study used evidence from a recent randomized controlled trial of patients with intermediate- and high-risk bladder cancer, the BOXIT trial (bladder COX-2 [cyclo-oxygenase-2] inhibition trial).

## Materials and Methods

### BOXIT Trial

The BOXIT trial (ISRCTN registry no. ISRCTN84681538; Cancer Research UK no. CRUK/07/004) was a randomized phase III placebo-controlled trial that evaluated the addition of celecoxib to standard treatment for patients with NMIBC and an intermediate or a high risk of recurrence. From 2007 to 2012, 472 patients with transitional cell carcinoma NMIBC were recruited. The patients had a mean age of 65.9 years, and most patients were men (79%). The median follow-up at the point of analysis was 44 months (interquartile range, 36-57 months). The trial found no clear treatment benefit for celecoxib, with no significant differences in the interval to the first recurrence of bladder cancer (ie, NMIBC or MIBC) between patients randomized to either celecoxib or placebo for 2 years. Further details of the study design, treatment schedules, patients, and clinical results from the trial have been previously reported.^12^

### Clinical Events

At trial entry, cases of intermediate- and high-risk NMIBC were defined according to the clinicopathologic features outlined by the European Association of Urology 2002 guidelines.^13^ The clinical events of interest during the trial were NMIBC recurrence and progression to MIBC. The grade and stage of NMIBC and MIBC were classified using the World Health Organization TNM classification.^14^ Patients could have experienced > 1 recurrence of NMIBC during the follow-up period. Disease progression was defined as the development of MIBC (stage ≥ pT2). Intermediate- and high-risk patients were recommended to undergo single adjuvant intravesical mitomycin C. The intermediate-risk patients were recommended to undergo 6 cycles of once-weekly adjuvant intravesical mitomycin C, and high-risk patients were recommended to undergo induction bacillus Calmette-Guérin with maintenance therapy for 3 years in accordance with international guidelines.^15^^,^^16^ Surveillance cystoscopy was performed at 3-month intervals for the first 2 years and then every 6 months for the third and fourth years. In the present report, we focused on the first 3 years of follow-up.

### HRQoL, Resource Use, and Cost Data

HRQoL was measured using the EQ-5D-3L, a generic, preference-based measure encompassing 5 dimensions of health (ie, mobility, self-care, usual activities, pain or discomfort, anxiety or depression) and an overall health rating, measured using a visual analog scale.^17^ The HRQoL values were generated using reported UK preference “tariffs” for the 243 health states described in the EQ-5D-3L.^18^ The values range from 1.0 (perfect health status) to −0.594, with 0 indicating death and negative values reflecting health states considered to be worse than death.^19^ The 346 high-risk patients in the trial completed scheduled EQ-5D self-assessments at baseline (trial entry) and at 2, 3, 6, 12, 24, and 36 months of follow-up. The 126 intermediate-risk patients completed scheduled EQ-5D self-assessments at baseline and at 12, 24, and 36 months of follow-up.

The cost analysis used resource use data from questionnaires collected during the trial and took the perspective of the National Health Service and personal social services. The relevant resources used were those related to the diagnosis, treatment, and 3-year follow-up data of the patients included in the BOXIT trial. These included endoscopic investigations, together with the primary, secondary, and palliative care data, and the therapeutic procedures used, including radical cystectomy, chemotherapy, radical radiotherapy, immunotherapy regimens, and intravesical therapy regimens. Missing information relating to the quantity or specific type of treatment administered after a clinical event was assumed to follow usual practice. The unit costs were obtained from a variety of sources (Supplemental Table 1 available in the online version) and inflated to 2017 prices.^20^ The costs of inpatient visits were determined using a fixed component relating to the first 2 days of stay, with a marginal component related to any additional days. Care was assumed to have been elective, unless stated otherwise. The total costs were aggregated into years after baseline, with each year estimated by multiplying the number of resources consumed during that period by their respective unit costs and summating.

The HRQoL analysis set consisted of the 316 high-risk patients who had fully completed ≥ 1 EQ-5D questionnaire(s) during the trial. The focus on high-risk patients was to use the most EQ-5D data available and provide the most interpretable estimates of effects, given the small number of MIBC and grade 3 NMIBC events in the intermediate-risk patients and the different EQ-5D follow-up schedules for the 2 risk groups. An analysis that included both risk groups with annual EQ-5D follow-up data was performed as a secondary analysis.

### Analysis Methods

The standard approach used to analyze HRQoL and cost data from clinical trials has been to compare these between treatment arms over time to calculate the quality-adjusted life-years (QALYs) and total costs for each patient in the trial.^21^ For trials showing no clinically or statistically significant benefit from a new treatment, this method will have little value. However, such trials offer a means of estimating the costs and HRQoL associated with a disease. This can include an exploration of how the HRQoL and costs vary between patients and how the patient characteristics and the clinical events they experience could explain some of this variation.^22^^,^^23^ Such analyses can provide valuable information to those assessing the potential value of other new treatments for similar patients.^24^

In the present study, 2 forms of analysis were conducted for both costs and HRQoL. The first analysis was descriptive, with the mean EQ-5D scores calculated at each follow-up period of interest, and the mean costs calculated annually. The patients were grouped in accordance with the types of events experienced during the 3-year follow-up period. The costs were categorized into resource-related groups for comparison. The second analysis was used to establish the effects of an event (ie, NMIBC, MIBC) on each outcome measure. The patients’ clinical events were linked to their closest post-event assessment. If multiple NMIBC recurrences had occurred between the EQ-5D or cost assessments, the recurrence with the highest grade was recorded. The effects of the events on the HRQoL and costs were computed using repeated measures regression, controlling for the relevant baseline covariates chosen on the basis of clinical relevance. These included: baseline HRQoL, randomized treatment, history of bladder cancer, patient characteristics (ie, age, body mass index, gender, diabetes), follow-up year, risk group, and interaction terms, as appropriate.

To evaluate the HRQoL and costs, separate generalized estimating equation models were implemented in accordance with reporting guidelines.^25^^,^^26^ The model fit, comparison, and selection of the working correlation structure was performed using the quasi-likelihood information criterion.^27^^,^^28^ Dependent variables of the annual costs and EQ-5D scores were assumed to follow the gamma and normal distributions, respectively.

## Results

### Patient Characteristics and Events

Patients experiencing disease recurrence and progression had characteristics similar to those of the patients without disease recurrence and progression. However, modest differences in the rates of diabetes and a history of NMIBC were noted (Table 1). We assessed whether systematic differences were present between patients with and without missing EQ-5D data at different follow-up points and found that the differences were small (Supplemental Tables 2 and 3 available in the online version). This finding supported the assumption from our complete case analysis that the occurrence of missing data was completely at random.

**Table 1:** Patient Characteristics

Characteristic	Total (n = 472)	High Risk (n = 346)	Intermediate Risk (n = 126)	No Event (n = 321)	Progression (n = 29)	Recurrence^a^			
						Overall (n = 138)	Grade 1 (n = 36)	Grade 2 (n = 62)	Grade 3 (n = 46)
EQ-5D, baseline	0.87 ± 0.15	0.86 ± 0.17	0.85 ± 0.22	0.88 ± 0.15	0.87 ± 0.13	0.87 ± 0.16	0.85 ± 0.20	0.91 ± 0.11	0.87 ± 0.14
Age, y	65.9 ± 9.9	65.8 ± 10.3	66.2 ± 8.8	65.7 ± 10.2	67.8 ± 7.1	66.2 ± 9.3	65.9 ± 10.3	66.1 ± 7.8	68.0 ± 7.7
BMI, kg/m^2^	27.8 ± 4.6	27.9 ± 4.6	27.7 ± 4.5	27.8 ± 4.3	27.0 ± 4.2	28.1 ± 5.2	27.8 ± 6.5	28.7 ± 5.5	27.9 ± 4.6
Male gender	374 (79.2)	278 (80.3)	96 (76.2)	262 (81.6)	25 (86.2)	102 (73.9)	27 (75.0)	45 (72.6)	33 (71.7)
Diabetes	42 (8.9)	30 (8.7)	12 (9.6)	23 (7.2)	2 (6.9)	19 (13.8)	6 (16.7)	8 (12.9)	8 (17.4)
NMIBC history	159 (34.0)	95 (27.8)	64 (51.2)	94 (29.7)	14 (48.3)	58 (42.3)	17 (47.2)	30 (48.4)	16 (35.6)
Celecoxib	236 (50.0)	167 (48.3)	69 (54.8)	164 (51.1)	13 (44.8)	65 (47.1)	22 (61.1)	30 (48.4)	17 (37.0)
Smoking status									
Never	145 (39.6)	113 (33.0)	32 (25.8)	101 (31.8)	8 (28.6)	42 (30.9)	10 (2.8)	16 (26.2)	18 (40.0)
Previous	252 (54.1)	187 (54.7)	65 (52.4)	173 (54.4)	16 (57.1)	70 (51.5)	19 (52.8)	34 (55.7)	21 (46.7)
Current	69 (14.8)	42 (12.3)	27 (21.8)	44 (13.8)	4 (14.3)	24 (17.7)	7 (19.4)	11 (18.0)	6 (13.3)
ECG result									
Normal	370 (78.6)	276 (79.8)	94 (75.2)	250 (78.1)	24 (82.8)	109 (79.0)	8 (77.8)	49 (79.0)	37 (80.4)
Abnormal	101 (21.4)	70 (20.2)	31 (24.8)	70 (21.9)	5 (17.2)	29 (21.0)	28 (77.8)	13 (20.1)	9 (19.6)

Data presented as mean ± standard deviation or n (%).

Abbreviations: ECG = electrocardiogram; NMIBC = non–muscle-invasive bladder cancer.

a The number of patients experiencing a recurrence exceeded the sum of graded recurrences because of missing grade data and patients experiencing multiple recurrences of different grades.

The recurrence of NMIBC was > 8 times more common than was progression to MIBC. A total of 233 cases of NMIBC recurrence in 138 patients (29.2%; of all 472 patients) had been recorded during the 3-year follow-up period. In contrast, 29 patients (6.1%) had experienced progression to MIBC (62.1% had undergone subsequent radical surgery). Of the 233 recurrent NMIBC events, 37 were not graded, 46 (9.7%) had experienced ≥ 1 grade 3 NMIBC recurrence (32.6% had undergone subsequent radical surgery), and 62 (13.1%) and 36 (7.6%) patients, respectively, had experienced ≥ 1 grade 2 and grade 1 recurrences (jointly, 4.1% had undergone subsequent radical surgery). Further details of the clinical events in the trial are provided in Supplemental Table 4 (available in the online version).

### HRQoL Analysis

**Figure 1:**
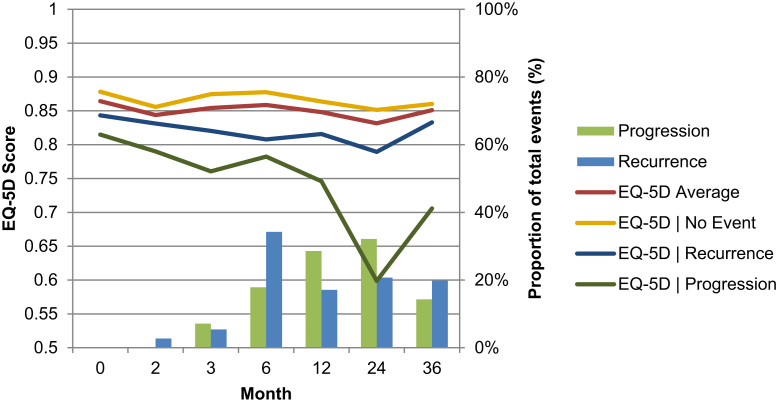
EQ-5D Scores for High-risk Patients for Each Event-related Subgroup and Associated Proportion of Events in Each Follow-up Point During 3 Years of Follow-up. The *x*-Axis Represents Time in Months After Baseline With Categories and Their Distance Solely Indicative of Trial Follow-up and Not Equating to the Length of Time Between Intervals

The completion rate of the EQ-5D during the 3-year period was 79% (range, 58%-84%) across the points of follow-up. The completion rates after a NMIBC recurrence and progression to MIBC were 60% and 38%, respectively. An overview of the observed mean EQ-5D index scores for high-risk patients and the proportion of events occurring between each EQ-5D follow-up period are presented in Figure 1. Full details of the HRQoL descriptive results are provided in Supplemental Tables 5 and 6 (available in the online version).

**Supplemental Figure 1:**
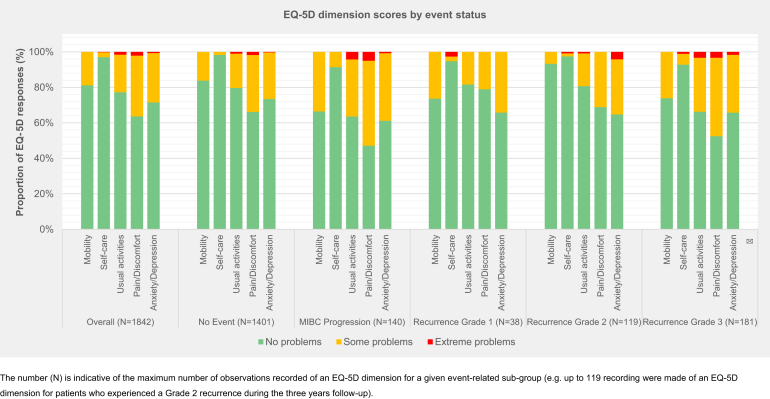
EQ-5D Responses Stratified by Dimension and Severity Level for High-risk Patients for Each Event-related Subgroup During 3 Years of Follow-up. The Number Indicates the Maximum Number of Observations Recorded of an EQ-5D Dimension for a Given Event-related Subgroup (eg, ≤ 119 Recordings Were Made of an EQ-5D Dimension for Patients Who Had Experienced a Grade 2 Recurrence During the 3-year Follow-up Period)

The set of subgroups comprising patients who had experienced ≥ 1 of the specified clinical events during the 3-year follow-up period or had experienced no event is presented in Figure 1. These findings suggest that NMIBC recurrence and MIBC progression could be associated with deterioration in HRQoL at specific points. The variation in HRQoL at specific follow-up points was largely driven by the events experienced by the patients. In contrast, the variation in HRQoL between the follow-up points was related to the underlying within-patient variations, the nonuniform distribution of events over time, and the sampling error, exacerbated by partitioning modestly sized subgroups. A comparison of the EQ-5D dimensions stratified by the event-related subgroup found greater proportions of patients reporting problems with pain or discomfort and undertaking usual activities when experiencing a grade 3 recurrence or MIBC progression compared with no events during the 3-year follow-up period (Supplemental Figure 1 available in the online version).

The statistically significant effects of clinical events on HRQoL in terms of the estimated decrements and mean health-state values are listed in Table 2. Progression to MIBC and NMIBC grade 3 recurrence were associated with predicted mean decrements in HRQoL of −0.10 (95% confidence interval, −0.17 to −0.03) and −0.08 (95% confidence interval, −0.13 to −0.03), respectively (*P* < .01). In contrast, the recurrence of NMIBC grade 1 and grade 2 was associated with positive, but statistically insignificant (*P* > .1), increments in HRQoL compared with patients without cancer.

**Table 2:** Estimated Statistically Significant Effects on HRQoL and Associated Health State Values From Clinical Events (High-risk Patients Only)

Variable	Estimated HRQoL Decrement^a^	Estimated Health State Value^a^
No event	NA	0.84606 (0.83292 to 0.85921)
NMIBC recurrence (grade 3)	−0.08306^b^ (−0.13379 to −0.03233)	0.76300 (0.71178 to 0.81422)
MIBC progression	−0.09909^b^ (−0.17256 to −0.02561)	0.74698 (0.67309 to 0.82087)

Data presented as mean (95% confidence interval).

Abbreviations: HRQoL = health-related quality of life; MIBC = muscle-invasive bladder cancer; NA = not applicable; NMIBC = non–muscle-invasive bladder cancer.

a Multivariate HRQoL longitudinal model controlled for baseline EQ-5D score, treatment (celecoxib), patient characteristics, bladder cancer history, annual time dummies, and events.

b *P* < .01.

The secondary analysis showed that introducing an interaction term into the regression analysis revealed that patients with grade 3 NMIBC recurrence in the first year experienced larger decrements in HRQoL (−0.11) compared with those with recurrence in subsequent years (−0.04). The small numbers precluded the same analysis for MIBC progression. Including both high- and intermediate-risk patients in the analysis using only the annual EQ-5D assessment data generated NMIBC recurrence estimates closer to 0 for all grades, with only MIBC events resulting in a statistically significant decrement in HRQoL (*P* < .05). Irrespective of the bladder cancer grade or stage, radical cystectomy was associated with a −0.17 decrement in HRQoL. All regression results and primary variance–covariance matrices are presented in Supplemental Table 10, Supplemental Table 11, Supplemental Table 12, Supplemental Table 13, Supplemental Table 7, Supplemental Table 8, Supplemental Table 9 (available in the online version).

### Cost Analysis

**Figure 2:**
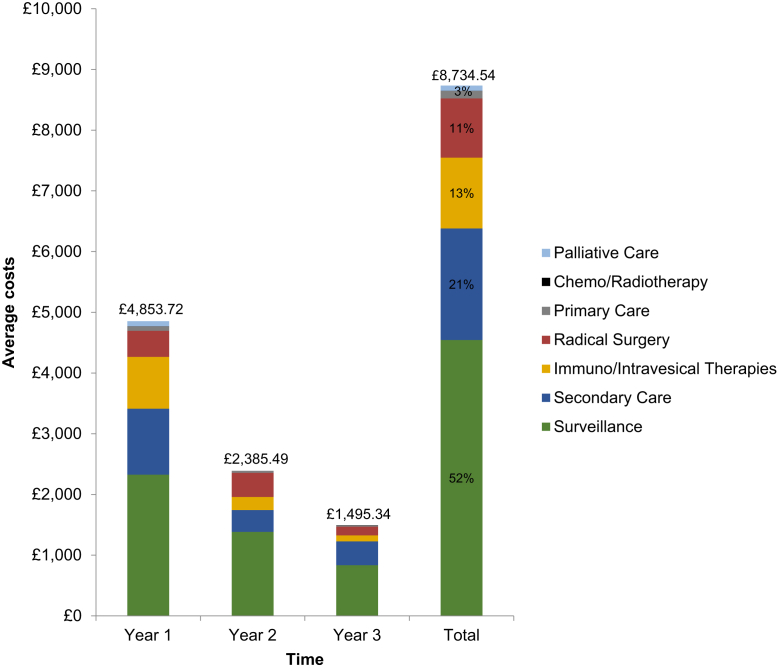
Mean Costs per Patient Over Time Stratified by Resource Category for Intermediate- and High-risk Patients

The mean costs per patient for each type of care (Supplemental Table 1 available in the online version), annually and in total, are reported in Figure 2. The mean cost of treatment for a patient with NMIBC was £4854 in the first year, with a total cost of £8735 over three years. These results suggest that the costs decline over time, with mean costs of £1496 in year 3. Endoscopic surveillance was the principal cost driver, accounting for > 52% of the total costs and representing a high proportion in years 2 (£1384 of £2386) and 3 (£835 of £1496). These estimates resulted in a 3-year total cost for the UK NMIBC bladder cancer cohort diagnosed in 2015 at ~£66.14 million, assuming that 74.5% of the 10,171 UK bladder cancer cases were NMIBC.^2^^,^^29^

**Figure 3:**
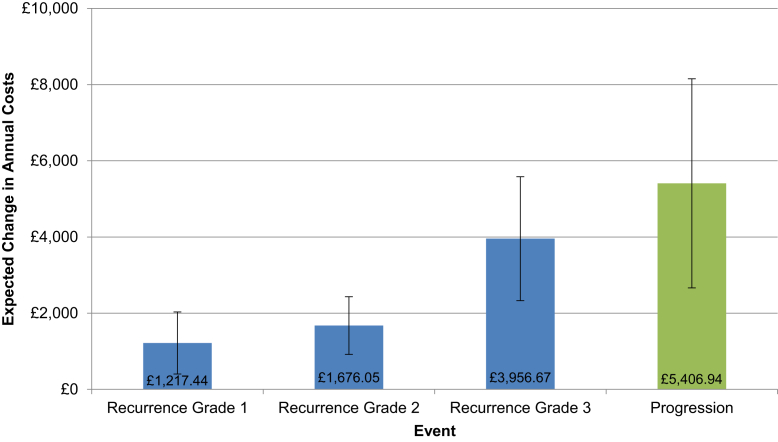
Estimated Mean Change in Annual Cost per Patient Associated With Clinical Events (95% Confidence Intervals Shown by Vertical Bars) From a Multivariate Longitudinal Panel Cost-related Analysis Controlling for Treatment, Patient Characteristics, Risk Group, Annual Time Dummies, Bladder Cancer Events, and Interactions

The effect of the clinical events on annual costs is shown in Figure 3, which indicated that MIBC progression and all grades of NMIBC recurrence led to increased costs. Higher grades of NMIBC were associated with higher costs, with grade 3 recurrence events necessitating more intensive therapy and closer surveillance. Progression to MIBC was associated with the greatest cost increment, with a £5407 increase in the expected annual cost per patient, again reflecting the more intensive therapy. Additionally, the treatment and surveillance of high-risk patients were associated with a £1968 increase in mean costs in the first year, although the costs had declined to £457 and £74 in years 2 and 3, respectively. The predicted mean costs per patient by year, event status, and risk group are shown in Table 3.

**Table 3:** Estimated Patient Costs Across Time, Risk Group, and Event Status^a^

Risk Group	Year	No Bladder Cancer	NMIBC Recurrence			MIBC Progression
			Grade 1	Grade 2	Grade 3	
High						
	1	£4796	£6014	£6472	£8753	£10,374
	2	£2363	£3581	£4039	£6320	£7940
	3	£1387	£2605	£3063	£5344	£6964
Intermediate						
	1	£2828	£4046	£4505	£6785	£8406
	2	£1907	£3125	£3583	£5864	£7484
	3	£1314	£2532	£2990	£5271	£6891

Abbreviations: HRQoL = health-related quality of life; MIBC = muscle-invasive bladder cancer; NA = not applicable; NMIBC = non–muscle-invasive bladder cancer.

a Predicted values from a multivariate longitudinal panel cost-related analysis controlling for treatment, patient characteristics, risk group, annual time dummies, bladder cancer events, and interactions.

## Discussion

Published economic evaluations of treatments for bladder cancer have lacked robust estimates of clinical effects on HRQoL and costs.^30^^,^^31^ Furthermore, clinicians should understand the consequences of clinical events on patients’ well-being and the health service costs. The present study has provided new evidence on the costs and HRQoL associated with NMIBC occurrence, recurrence, and progression to MIBC, supporting future clinical and economic evaluations. Our findings suggest that NMIBC will have an average cost of £8735 during a 3-year period, with cases of grade 1, 2, and 3 NMIBC recurrences and progression to MIBC associated with £1218, £1677, £3957, and £5407 increases in annual costs, respectively. In addition, grade 3 recurrence and progression to MIBC were associated with statistically significant decrements in HRQoL (−0.08 and −0.10, respectively).

Singer et al^32^ reported that patients with bladder cancer, whether muscle invasive or not, will experience significant and clinically relevant deteriorations in HRQoL. Little evidence has contradicted the idea that patients with MIBC will experience a significant health burden; however, the same cannot necessarily be said for those with NMIBC. The commonly reported NMIBC morbidities have included mental health effects at diagnosis, physical discomfort, sexual problems, and urinary symptoms.^33^, ^34^, ^35^ However, these have rarely translated into reductions in longer term health outcomes and, in some cases, have not been recorded at all.^9^^,^^36^ It has been suggested that patients might become “accustomed” to NMIBC and its related management, accepting recurrences as a part of their lives.^10^ The evidence presented from the BOXIT trial has offered some additional support for this view, but suggests that not all cases of NMBIC recurrence should be considered equal. Based on the recommended NMIBC surveillance guidelines, our results suggest that the negative effect of a NMIBC recurrence on HRQoL will be concentrated within the high-grade strata (grade 3), especially in the first year after the diagnosis. Furthermore, no evidence of negative HRQoL outcomes from grade 1 or 2 NMIBC recurrences was found. This might, at least in part, be explained by the low rates of radical surgery observed for grade 1 and 2 NMIBC recurrences. The results from supplementary analyses have supported these findings, with the use of cystectomy a large and significant predictor of HRQoL status. In addition, the patient groups with the highest rates of radical surgery (for grade 3 recurrence and progression) were most likely to report related problems with pain or discomfort and undertaking usual activities. A fuller understanding of the mechanisms behind these findings requires further prospective research.

Sangar et al,^37^ estimated that the UK cost in 2001 to 2002 for the diagnosis, treatment, and 5-year follow-up of each bladder cancer case was £55.39 million, at a mean cost of £8349.20. Allowing for inflation and the different follow-up periods, their results are similar to those from the present study. To put this into context, it would be less costly per patient to treat stage 2 colon, rectal, and non–small-cell lung cancer in the United Kingdom.^38^ The results from our analysis compliment those from the earlier study, showing the prominent role of endoscopic surveillance in driving the costs, which has remained the primary target for innovation in bladder cancer management.^5^^,^^39^^,^^40^ Optimizing surveillance has also remained a research priority. Less costly and noninvasive urinary biomarkers represent an attractive option; however, to date, no commercially available test has the diagnostic accuracy to replace cystoscopy because patients and physicians require a test with high sensitivity before widespread acceptance.^41^, ^42^, ^43^ Similar to others, we found that progression to MIBC will be associated with higher costs for intermediate- and high-risk patients.^44^

The relatively large sample size, prospective study design, and the use of a validated HRQoL instrument represent the strengths of the present study. To the best of our knowledge, this is the first study to estimate both the mean and the marginal HRQoL and the cost effects across multiple grades and stages of bladder cancer. However, the present study had several important limitations. Despite the BOXIT protocol remaining representative of current UK guidelines (other than celecoxib treatment), differences between the BOXIT trial and current clinical practice have occurred (eg, the European Association of Urology now recommends bacillus Calmette-Guérin instillations for intermediate-risk patients and have revised the definitions of risk^45^). In addition, the trial’s exclusion criteria could have limited the generalizability of our study, with results applicable to a cohort healthier than what might be observed in clinical practice. With respect to HRQoL, the EQ-5D is a generic measure of health outcomes suitable to assess the value of healthcare interventions across different disease areas. The EQ-5D is the preferred instrument of the National Institute for Health and Care Excellence for cost-effectiveness analysis. Although the measure showed important differences between the patient subgroups, further research could assess whether the EQ-5D is sufficiently sensitive to detect important clinical changes in patients with bladder cancer. Regarding the study findings, the true negative repercussions of MIBC might differ from those reported because the number of patients who progressed to MIBC was relatively small because BOXIT trial was powered to investigate the interval to the first recurrence. This, coupled with the low postprogression EQ-5D response rate, resulted in uncertain estimates and might lead to overestimates of the HRQoL because patients with relatively poor health outcomes after the development of MIBC might be less likely to complete the EQ-5D. Moreover, the increasingly protracted EQ-5D follow-up periods meant that the clinical events in the study became progressively distant from completion of the EQ-5D. Whether improvements in the reported postevent HRQoL outcomes over time stemmed from the true underlying dynamics of bladder cancer or had resulted only from time-related disparities between the event and the follow-up evaluation remains to be determined.

The costs could have been underestimated for several reasons. First, our analysis of the effect of the events on the annual costs neglected the potential dynamics and spillover effects between the evaluation periods. Bladder cancer events will inevitably prompt immediate resource use; however, the costs incurred from stricter surveillance and the greater risk of related events will be realized further into the future. Understanding these dynamics requires a more detailed collection of the resource use data and remains a potential avenue for further research. Second, the assumption that the treatments were elective could, again, have underrepresented the costs. Third, the protracted and persistent nature of bladder cancer has far broader cost effects than those incurred only by the NHS within 3 years. A wider perspective would give a more comprehensive account of the earnings, productivity, and time lost by patients with bladder cancer and their informal caregivers.

## Conclusion

The results from our analysis of the BOXIT trial data suggest that patients with NMIBC will experience decrements in HRQoL, with significant costs imposed in the event of disease recurrence or progression, and the costs increasing with the abnormality and invasiveness of the lesion.

### Clinical Practice Points

• A need exists to evaluate the costs and HRQoL implications of bladder cancer and its recurrence and progression to assess the disease burden, inform resource allocation decisions, and aid further research.
• It has been shown that NMIBC will be associated with considerable costs in the United Kingdom and that patients will experience significant decrements in HRQoL with progression to MIBC; however, the effect from NMIBC recurrence is less clear.
• In our study, we reported both the mean and the marginal UK HRQoL and cost effects across multiple grades and stages of bladder cancer for patients with NMIBC and found significant decrements in HRQoL related to grade 3 recurrence and progression to MIBC; the cost effects increased with the lesion's abnormality and invasiveness.
• The evidence presented from the BOXIT trial suggests that not all cases of NMBIC recurrence should be considered equal with respect to the effects on patient HRQoL or the consequent healthcare costs.
• The results from the present study could help to lay the foundation for future related burden of disease studies and cost-effectiveness analyses.

## Disclosure

E.H. reports grants from 10.13039/501100000289Cancer Research UK, grants from Kyowa Hakko UK, grants from Alliance Pharma (previously Cambridge Laboratories Ltd), nonfinancial support from Pfizer Inc, during the conduct of the study, grants from Pfizer Inc, grants and nonfinancial support from Merck Sharp & Dohm, grants and nonfinancial support from Astra Zeneca, grants from Janssen-Cilag, grants and nonfinancial support from Bayer, grants from Aventis Pharma Ltd (Sanofi), and grants from Accuray Inc. M.S. reports grants from 10.13039/501100000289Cancer Research UK (CRUK/07/04) and educational grants from Kyowa Hakko UK Ltd and Cambridge Laboratories Ltd during the conduct of the study. The remaining authors declare that they have no competing interests.

## Figures and Tables

**Supplemental Table 1:** Unit Costs

Care	Unit Costs^a^	Source
Primary care		PSSRU health and social care, 2017^20^
GP home visit	£86	
Specialist nurse home visit	£57	
General practice surgery visit, GP	£32	
General practice surgery visit (nurse)	£10	
Secondary care		NHS schedule reference costs 2016-2017^46^
Outpatient attendance	£108	TOA: urology outpatient attendance (service code, 101)
Inpatient attendance	£820	EL: minor bladder procedures, age ≥ 19 y (HRG code, LB15E)
Inpatient excess days	£397	EL XS: intermediate open bladder procedures (HRG code, LB12Z)
Palliative care^b^	£12,968	NICE technology assessment January 2010^47^
Surveillance		NICE technology assessment January 2010^47^
Flexible cystoscopy	£449	
Rigid cystoscopy	£1176	
Intravesical/immunotherapy		
Mitomycin instillation	£80	British National Formulary 2018
Bacillus Calmette-Guérin instillation	£101	NICE technology assessment January 2010^47^
Radical surgery		
Cystectomy	£9973	Total HRGs: cystectomy with urinary diversion and reconstruction (code, LB39C/LB39D)
Lobectomy	£6601	NICE clinical guideline 121 (2011)^48^
Nephroureterectomy	£6471	Complex, open or laparoscopic, kidney or ureter procedures, with CC score 0-1 (HRG code, LB60F)
Renogram	£256	Renogram, age ≥ 19 y (HRG code, RN25A)
Chemotherapy/radiotherapy^c^		
Radical radiotherapy	£1156	NICE technology assessment January 2010^47^
Gemcitabine, cisplatin	£169	eMit drug unit costs and London Cancer Network administration schedules
Gemcitabine, carboplatin	£232	eMit drug unit costs and London Cancer Network administration schedules
5-FU, MMC	£104	eMit drug unit costs and London Cancer Network administration schedules
Carboplatin, etoposide	£173	eMit drug unit costs and London Cancer Network administration schedules

Abbreviations: 5-FU = 5-fluorouracil; CC = complexity and comorbidity; EL = elective inpatient; EL XS = elective inpatient excess bed days; eMIT = electronic market information tool; GP = general practitioner; HRGs = healthcare resource groups; HRQoL = health-related quality of life; MIBC = muscle-invasive bladder cancer; MMC = mitomycin; NA = not applicable; NHS = National Health Service; NICE = National Institute for Health and Care Excellence; NMIBC = non–muscle-invasive bladder cancer; PSSRU = Personal Social Service Research Unit; TOA = total outpatient attendance.

a Inflated to 2017 prices using the PSSRU hospital and community health services index; presented costs were rounded up to the nearest pound sterling.

b Duration of 135 days used in accordance with reference material and per day NHS schedule reference for 2016-2017 costs applied.

c Specific chemotherapy unit costs were calculated as the product of the specific drug costs (using eMit), dosage, and observed/recommended number of cycles (recommended schedules from the NHS Cancer Network were used if trial information was missing).

**Supplemental Table 2:** Summary Statistics Comparison: Missing and Nonmissing EQ-5D Data Collection^a^

Variable	Missing Values		Nonmissing Values	
	n	Mean	n	Mean
Month 2				
Age	187	65.19	285	66.38
BMI	180	27.71	266	27.89
Gender	187	75%	285	82%
Never smoked	48	26%	97	34%
Previous smoker	97	53%	155	55%
Current smoker	39	21%	30	11%
ECG result	186	23%	285	21%
Celecoxib	187	52%	285	48%
Diabetes	186	11%	285	8%
History	183	44%	284	27%
Month 3				
Age	185	65.41	287	66.26
BMI	179	27.77	267	27.85
Gender	185	75%	287	82%
Never smoked	44	24%	101	35%
Previous smoker	97	53%	155	55%
Current smoker	41	23%	28	10%
ECG result	184	21%	287	22%
Celecoxib	185	54%	287	47%
Diabetes	184	11%	287	8%
History	181	46%	286	27%
Month 6				
Age	196	65.27	276	66.40
BMI	189	27.66	257	27.93
Gender	196	74%	276	83%
Never smoked	50	26%	95	35%
Previous smoker	103	53%	149	55%
Current smoker	40	21%	29	10%
ECG result	195	21%	276	22%
Celecoxib	196	53%	276	48%
Diabetes	195	12%	276	7%
History	192	46%	275	26%
Month 12				
Age	125	65.67	347	66.02
BMI	120	27.91	326	27.78
Gender	125	78%	347	80%
Never smoked	31	25%	114	33%
Previous smoker	66	54%	186	54%
Current smoker	25	21%	44	13%
ECG result	124	19%	347	22%
Celecoxib	125	52%	347	49%
Diabetes	124	9%	347	9%
History	122	34%	345	34%
Month 24				
Age	163	66%	309	66.03
BMI	155	27.65	291	27.91
Gender	163	79%	309	80%
Never smoked	42	26%	103	34%
Previous smoker	90	56%	162	53%
Current smoker	28	18%	41	13%
ECG result	162	16%	309	24%
Celecoxib	163	52%	309	49%
Diabetes	162	10%	309	8%
History	160	36%	307	33%
Month 36				
Age	191	66.21	281	65.73
BMI	183	27.62	263	27.95
Gender	191	79%	281	79%
Never smoked	47	25%	98	35%
Previous smoker	100	53%	152	55%
Current smoker	41	22%	28	10%
ECG result	190	20%	281	22%
Celecoxib	191	53%	281	48%
Diabetes	190	9%	281	9%
History	188	36%	279	33%

Abbreviations: BMI = body mass index; ECG = electrocardiography; EQ-5D = EuroQoL 5 dimension.

a Patient gender (female = 0; male = 1); ECG result (normal = 1; abnormal = 0); Celecoxib (placebo arm = 0; treatment arm = 1); history (no history of NMIBC = 0; history of NMIBC = 1); diabetes (no diabetes = 0; diabetes = 1).

**Supplemental Table 3:** Summary Statistics Comparison: Missing and Nonmissing Costs^a^

Variable	Missing Values		Nonmissing Values	
	n	Mean	n	Mean
Year 1				
Age	25	69.16	447	65.75
BMI	23	27.29	423	27.85
Gender	25	80%	447	79%
Never smoked	6	24%	145	32%
Previous smoker	13	52%	239	53%
Current smoker	6	24%	63	14%
ECG result	24	8%	447	22%
Celecoxib	25	52%	447	50%
Diabetes	24	13%	442	9%
History	23	52%	444	33%
Year 2				
Age	30	68.57	442	65.75
BMI	28	26.91	418	27.88
Gender	30	80%	442	79%
Never smoked	7	23%	144	33%
Previous smoker	19	63%	223	53%
Current smoker	4	13%	65	15%
ECG result	29	14%	442	22%
Celecoxib	30	47%	442	50%
Diabetes	29	10%	442	9%
History	28	39%	439	34%
Year 3				
Age	47	68.09	425	65.69
BMI	45	27.15	401	27.89
Gender	47	74%	425	80%
Never smoked	11	23%	140	33%
Previous smoker	26	55%	226	53%
Current smoker	10	21%	59	14%
ECG result	46	11%	425	23%
Celecoxib	47	43%	425	50%
Diabetes	46	7%	425	9%
History	45	42%	422	33%

Abbreviations: BMI = body mass index; ECG = electrocardiography.

a Patient gender (female = 0; male = 1); ECG result (normal = 1; abnormal = 0); Celecoxib (placebo arm = 0; treatment arm = 1); history (no history of NMIBC = 0; history of NMIBC = 1); diabetes (no diabetes = 0; diabetes = 1).

**Supplemental Table 4:** Trial Events^a^

Variable	Trial Event, n													
	Month 2		Month 3		Month 6		Month 12		Month 24		Month 36		Total Events	Total Patients
	HR	IR	HR	IR	HR	IR	HR	IR	HR	IR	HR	IR		
MIBC progression	0	0	2	0	5	0	8	0	9	1	4	0	29	29
NMIBC recurrence	3	2	6	6	38	20	19	32	23	44	22	18	233	138
Recurrence grade	2	2	6	4	35	14	16	29	17	37	17	17	196	121
Unknown	0	0	0	0	0	0	2	0	0	0	1	0	3	3
Grade 1	0	1	0	0	4	7	3	11	5	14	0	9	54	36
Grade 2	1	0	3	4	9	7	4	15	7	21	7	7	85	62
Grade 3	1	1	3	0	22	0	7	3	5	2	9	1	54	46

Abbreviations: EQ-5D = EuroQoL 5 dimension; HR = high-risk (patients); IR = intermediate-risk (patients); MIBC = muscle-invasive bladder cancer; NMIBC = non–muscle-invasive bladder cancer.

a For cases in which multiple NMIBC recurrences had developed between the EQ-5D and/or annual cost assessments, the analysis set was applied to the recurrence with the highest grade recorded (see the “Materials and Methods” section for details).

**Supplemental Table 5:** Observed EQ-5D Scores From the BOXIT Trial for High-risk Patients

EQ-5D	EQ-5D Event-Specific Scores						
	Baseline	Month 2	Month 3	Month 6	Month 12	Month 24	Month 36
Average							
Mean ± SD	0.86 ± 0.17	0.84 ± 0.20	0.85 ± 0.18	0.86 ± 0.18	0.85 ± 0.19	0.83 ± 0.19	0.85 ± 0.19
Patients, n	309	284	286	274	250	223	205
No event							
Mean ± SD	0.88 ± 0.15	0.86 ± 0.20	0.87 ± 0.15	0.88 ± 0.16	0.86 ± 0.17	0.85 ± 0.16	0.86 ± 0.18
Patients, n	224	210	209	209	297	181	168
Progression							
Mean ± SD	0.82 ± 0.23	0.79 ± 0.23	0.76 ± 0.22	0.78 ± 0.26	0.75 ± 0.26	0.60 ± 0.30	0.71 ± 0.35
Patients, n	28	26	28	19	15	10	7
Recurrence							
Mean ± SD	0.84 ± 0.20	0.83 ± 0.20	0.82 ± 0.21	0.81 ± 0.21	0.82 ± 0.21	0.79 ± 0.25	0.83 ± 0.20
Patients, n	71	62	64	56	45	35	33
Recurrence grade							
1							
Mean ± SD	0.90 ± 0.11	0.85 ± 0.12	0.88 ± 0.14	0.86 ± 0.11	0.93 ± 0.20	0.81 ± 0.21	0.83 ± 0.33
Patients, n	8	7	8	7	4	4	4
2							
Mean ± SD	0.88 ± 0.14	0.90 ± 0.10	0.89 ± 0.13	0.86 ± 0.20	0.84 ± 0.14	0.70 ± 0.32	0.78 ± 0.78
Patients, n	23	21	21	20	17	14	10
3							
Mean ± SD	0.85 ± 0.17	0.82 ± 0.21	0.79 ± 0.23	0.75 ± 0.22	0.79 ± 0.26	0.77 ± 0.27	0.80 ± 0.22
Patients, n	36	31	33	27	20	16	6

Abbreviations: BOXIT = bladder COX-2 (cyclooxygenase-2) inhibition trial; EQ-5D = EuroQoL 5 dimension; SD = standard deviation.

**Supplemental Table 6:** Observed EQ-5D Scores From the BOXIT Trial for Intermediate- and High-Risk Patients

EQ-5D	EQ-5D Event-Specific Scores			
	Baseline	Month 12	Month 24	Month 36
Average				
Mean ± SD	0.86 ± 0.19	0.85 ± 0.20	0.83 ± 0.20	0.85 ± 0.20
Patients, n	410	347	309	281
No event				
Mean ± SD	0.87 ± 0.16	0.86 ± 0.18	0.84 ± 0.18	0.85 ± 0.20
Patients, n	275	244	224	209
Progression				
Mean ± SD	0.82 ± 0.23	0.71 ± 0.28	0.66 ± 0.55	0.71 ± 0.35
Patients, n	29	16	11	7
Recurrence				
Mean ± SD	0.85 ± 0.21	0.84 ± 0.23	0.84 ± 0.24	0.87 ± 0.19
Patients, n	121	95	78	68
Recurrence grade				
1				
Mean ± SD	0.81 ± 0.29	0.77 ± 0.31	0.80 ± 0.30	0.87 ± 0.26
Patients, n	28	24	21	18
2				
Mean ± SD	0.91 ± 0.12	0.88 ± 0.16	0.84 ± 0.26	0.88 ± 0.19
Patients, n	54	48	41	33
3				
Mean ± SD	0.86 ± 0.17	0.80 ± 0.27	0.77 ± 0.29	0.83 ± 0.21
Patients, n	41	26	21	20

Abbreviations: BOXIT = bladder COX-2 (cyclooxygenase-2) inhibition trial; EQ-5D = EuroQoL 5 dimension; SD = standard deviation.

**Supplemental Table 7:** Primary HRQoL Regression

Variable	Coefficient	SE	z	*P* > z	95% CI
EQ5D score baseline	0.5967924	0.0406532	14.68	.000	0.5171136 to 0.6764713
Patient gender	0.0521357	0.0179587	2.90	.004	0.0169372 to 0.0873341
Age category, y					
50-59	−0.0187366	0.0338184	−0.55	.580	−0.0850194 to 0.0475462
60-69	−0.0039931	0.0318242	−0.13	.900	−0.0663674 to 0.0583812
70-79	−0.0108462	0.0331665	−0.33	.744	−0.0758513 to 0.0541589
> 80	−0.0243156	0.0396575	−0.61	0.540	−0.1020428 to 0.0534116
BMI category					
Overweight	−0.0100358	0.0166613	−0.60	0.547	−0.0426913 to 0.0226198
Obese	−0.0069584	0.0182376	−0.38	0.703	−0.0427036 to 0.0287867
Morbidly obese	−0.0652968	0.0651534	−1.00	0.316	−0.1929952 to 0.0624015
Smoking status					
Previous	−0.0033888	0.0148166	−0.23	0.819	−0.0324288 to 0.0256512
Current	−0.0069576	0.0239007	−0.29	0.771	−0.0538022 to 0.039887
ECG result	−0.0057031	0.016846	−0.34	0.735	−0.0387207 to 0.0273145
Celecoxib treatment	−0.0010674	0.0136832	−0.08	0.938	−0.0278859 to 0.0257511
Diabetes	−0.0989409	0.0252237	−3.92	0.000	−0.1483784 to −0.0495034
TCC history	−0.015477	0.0155777	−0.99	0.320	−0.0460086 to 0.0150547
Year					
2	−0.0250881	0.0103193	−2.43	0.015	−0.0453134 to −0.0048627
3	−0.0107493	0.0102873	−1.04	0.296	−0.0309119 to 0.0094134
Tumor recurrence					
Unknown	0.0334809	0.0823301	0.41	0.684	−0.1278831 to 0.1948449
Grade 1	0.062 0308	0.0555815	1.12	0.264	−0.046907 to 0.1709685
Grade 2	0.0518003	0.0339202	1.53	0.127	−0.014682 to 0.1182826
Grade 3	−0.0830612	0.0258832	−3.21	0.001	−0.1337914 to −0.0323311
Progression	−0.0990853	0.037488	−2.64	0.008	−0.1725605 to −0.0256102
Progression history	0.0043892	0.0516379	0.08	0.932	−0.0968193 to 0.1055976
Constant	0.3218822	0.0489321	6.58	0.000	0.225977 to 0.4177873

Abbreviations: BMI = body mass index; CI = confidence interval; ECG = electrocardiography; EQ-5D = EuroQoL 5 dimension; HRQoL = health-related quality of life; SE = standard error; TCC = transitional cell carcinoma.

**Supplemental Table 8:** Primary HRQoL Regression Including Time and Event Interaction

EQ-5D Score	Coefficient	SE	z	*P* > z	95% CI
EQ-5D score baseline	0.5973907	0.0407683	14.65	.000	0.5174862 to 0.6772952
Patient gender	0.0530437	0.0180207	2.94	.003	0.0177238 to 0.0883636
Age category, y					
50-59	−0.020057	0.0339243	−0.59	.554	−0.0865473 to 0.0464333
60-69	−0.0060109	0.03192 99	−0.19	.851	−0.0685923 to 0.0565705
70-79	−0.0113383	0.0332657	−0.34	.733	−0.0765379 to 0.0538613
>80	−0.0274407	0.0397877	−0.69	.490	−0.1054232 to 0.0505419
BMI category					
Overweight	−0.01043	0.0167119	−0.62	.533	−0.0431847 to 0.0223247
Obese	−0.0073803	0.0183151	−0.40	.687	−0.0432771 to 0.0285166
Morbidly obese	−0.0629669	0.0653087	−0.96	.335	−0.1909695 to 0.0650357
Smoking status					
Previous	−0.0029297	0.0148631	−0.20	.844	−0.0320609 to 0.0262014
Current	−0.0080374	0.0239813	−0.34	.738	−0.05504 to 0.0389651
ECG result	−0.0052007	0.0168996	−0.31	.758	−0.0383233 to 0.0279219
Celecoxib treatment	−0.0004786	0.0137258	−0.03	.972	−0.0273807 to 0.0264235
Diabetes	−0.1000132	0.0253062	−3.95	.000	−0.1496125 to −0.0504139
TCC history	−0.0157322	0.0156268	−1.01	.314	−0.0463602 to 0.0148957
Tumor recurrence & year interactions					
No cancer & > year 1	−0.0209844	0.0089722	−2.34	.019	−0.0385696 to −0.0033992
Unknown & year 1	0.0506889	0.1156377	0.44	.661	−0.1759568 to 0.2773346
Unknown & > year 1	0.0038289	0.1159498	0.03	.974	−0.2234286 to 0.2310864
Grade 1 & year 1	−0.028671	0.0896046	−0.32	.749	−0.2042929 to 0.1469509
Grade 1 & > year 1	0.0907741	0.0706822	1.28	.199	−0.0477605 to 0.2293087
Grade 2 & year 1	0.0284185	0.0468997	0.61	.545	−0.0635031 to 0.1203402
Grade 2 & > year 1	0.0690765	0.0486986	1.42	.156	−0.026371 to 0.1645239
Grade 3 & year 1	−0.1096423	0.0317785	−3.45	.001	−0.171927 to −0.0473577
Grade 3 & > year 1	−0.0429308	0.0428694	−1.00	.317	−0.1269532 to 0.0410917
Progression	−0.0937818	0.0373684	−2.51	.012	−0.1670225 to −0.0205412
Progression history	0.0053212	0.051364	0.10	.917	−0.0953503 to 0.1059927
Constant	0.3228122	0.0490795	6.58	.000	0.2266181 to 0.4190063

Abbreviations: BMI = body mass index; CI = confidence interval; ECG = electrocardiography; EQ-5D = EuroQoL 5-dimension; HRQoL = health-related quality of life; SE = standard error; TCC = transitional cell carcinoma.

**Supplemental Table 9:** HRQoL Regression With Intermediate- and High-Risk Patients and Annual EQ-5D

EQ-5D Score	Coefficient	SE	z	*P* > z	95% CI
EQ-5D score baseline	0.6222412	0.0440824	14.12	.000	0.5358413 to 0.7086411
Risk group	−0.0221777	0.0181653	−1.22	.222	−0.0577811 to 0.0134256
Patient gender	0.0344006	0.0192668	1.79	.074	−0.0033617 to 0.0721629
Age category, y					
50-59	−0.0421994	0.0414963	−1.02	.309	−0.1235307 to 0.039132
60-69	−0.051239	0.0393116	−1.30	.192	−0.1282882 to 0.0258103
70-79	−0.0600482	0.0411742	−1.46	.145	−0.1407482 to 0.0206518
>80	−0.0726505	0.0482603	−1.51	.132	−0.1672389 to 0.0219379
BMI category					
Overweight	−0.0221557	0.018897	−1.17	.241	−0.0591931 to 0.0148818
Obese	−0.0381121	0.0206114	−1.85	.064	−0.0785097 to 0.0022855
Morbidly obese	−0.10648	0.0743021	−1.43	.152	−0.2521095 to 0.0391495
Smoking status					
Previous	0.0102264	0.0171481	0.60	.551	−0.0233833 to 0.0438361
Current	−0.0547593	0.0252457	−2.17	.030	−0.1042399 to −0.0052786
ECG result	−0.0382036	0.0187138	−2.04	.041	−0.074882 to −0.0015251
Celecoxib treatment	−0.0081224	0.0155085	−0.52	.600	−0.0385186 to 0.0222737
Diabetes	−0.0627696	0.027287	−2.30	.021	−0.116251 to −0.0092881
TCC history	−0.0177708	0.016869	−1.05	.292	−0.0508335 to 0.0152919
Year					
2	−0.0205743	0.009907	−2.08	.038	−0.0399916 to −0.001157
3	−0.0187082	0.0106461	−1.76	.079	−0.0395742 to 0.0021579
Tumor recurrence					
Unknown	0.0616068	0.0957521	0.64	.520	−0.1260638 to 0.2492774
Grade 1	−0.005975	0.0317292	−0.19	.851	−0.068163 to 0.056213
Grade 2	0.0019765	0.0221878	0.09	.929	−0.0415108 to 0.0454638
Grade 3	−0.0434608	0.0277767	−1.56	.118	−0.0979022 to 0.0109806
Progression	−0.1020626	0.0428498	−2.38	.017	−0.1860467 to −0.0180785
Progression history	−0.0434159	0.0623099	−0.70	.486	−0.1655411 to 0.0787093
Constant	0.4007673	0.0596421	6.72	.000	0.2838709 to 0.5176637

Abbreviations: BMI = body mass index; CI = confidence interval; ECG = electrocardiography; EQ-5D = EuroQoL 5-dimension; HRQoL = health-related quality of life; SE = standard error; TCC = transitional cell carcinoma.

**Supplemental Table 10:** Base Case HRQoL Regression Including Cystectomy as a Covariate

EQ-5D Score	Coefficient	SE	z	*P* > z	95% CI
EQ-5D score baseline	0.636108	0.0377744	16.84	.000	0.5620715 to 0.7101444
Patient gender	0.0477877	0.0163828	2.92	.004	0.0156781 to 0.0798973
Age category, y					
50-59	−0.0288782	0.0346037	−0.83	.404	−0.0967001 to 0.0389438
60-69	−0.037565	0.0327225	−1.15	.251	−0.1016999 to 0.0265698
70-79	−0.0464312	0.0342036	−1.36	.175	−0.1134689 to 0.0206066
>80	−0.052182	0.0399786	−1.31	.192	−0.1305387 to 0.0261747
BMI category					
Overweight	−0.0177077	0.0156904	−1.13	.259	−0.0484604 to 0.0130449
Obese	−0.0150946	0.0172463	−0.88	.381	−0.0488967 to 0.0187074
Morbidly obese	−0.0273359	0.0608352	−0.45	.653	−0.1465707 to 0.0918989
Smoking status					
Previous	0.0064122	0.0142 637	0.45	.653	−0.0215443 to 0.0343686
Current	−0.0295427	0.0214073	−1.38	.168	−0.0715001 to 0.0124148
ECG result	−0.0215747	0.0157273	−1.37	.170	−0.0523997 to 0.0092503
Celecoxib treatment	−0.0081632	0.0129252	−0.63	.528	−0.0334961 to 0.0171696
Diabetes	−0.0881547	0.0233651	−3.77	.000	−0.1339494 to −0.0423601
TCC history	−0.022155	0.0137706	−1.61	.108	−0.049145 to 0.0048349
Year					
2	−0.0174281	0.0085872	−2.03	.042	−0.0342587 to −0.0005975
3	−0.0161801	0.0090078	−1.80	.072	−0.033835 to 0.0014749
Cystectomy	−0.1676828	0.0382576	−4.38	.000	−0.2426664 to −0.0926992
Constant	0.3340947	0.0476971	7.00	.000	0.24061 to 0.4275793

Abbreviations: BMI = body mass index; CI = confidence interval; ECG = electrocardiography; EQ-5D = EuroQoL 5-dimension; HRQoL = health-related quality of life; SE = standard error; TCC = transitional cell carcinoma.

**Supplemental Table 11:** Costing Regression

Total Costs	Coefficient	SE	z	*P* > z	95% CI
Tumor recurrence					
Unknown	1517.223	1729.041	0.88	.380	−1871.636 to 4906.082
Grade 1	1217.438	415.9633	2.93	.003	402.1653 to 2032.711
Grade 2	1676.051	385.9831	4.34	.000	919.5377 to 2432.564
Grade 3	3956.667	829.3751	4.77	.000	2331.122 to 5582.212
Risk group, high risk	1967.914	311.494	6.32	.000	1357.397 to 2578.431
Year					
2	−921.3536	251.7046	−3.66	.000	−1414.686 to −428.0217
3	−1514.189	233.9928	−6.47	.000	−1972.806 to −1055.571
Risk group & year					
High risk & year 2	−1511.85	343.8087	−4.40	.000	−2185.702 to −837.997
High risk & year 3	−1894.898	319.9745	−5.92	.000	−2522.036 to −1267.759
Progression	5406.938	1400.335	3.86	.000	2662.332 to 8151.544
Progression history	2269.138	806.8528	2.81	.005	687.7356 to 3850.54
TCC history	91.53518	91.50393	1.00	.317	−87.80923 to 270.8796
Patient gender	162.3912	104.348	1.56	.120	−42.12716 to 366.9096
Diabetes	−67.09895	147.0358	−0.46	.648	−355.2838 to 221.0859
Celecoxib treatment	−103.1504	90.55783	−1.14	.255	−280.6405 to 74.33965
Toxicity					
Mild condition	190.4007	173.7812	1.10	.273	−150.2041 to 531.0055
Moderate condition	171.735	300.5923	0.57	.568	−417.415 to 760.885
Celecoxib treatment & toxicity interaction					
Interaction					
1 & Mild condition	153.2397	242.295	0.63	.527	−321.6498 to 628.1292
1 & Moderate condition	390.0575	387.7738	1.01	.314	−369.9651 to 1150.08
Age, y					
50-59	36.85634	193.8437	0.19	.849	−343.0704 to 416.7831
60-69	62.7073	177.3931	0.35	.724	−284.9767 to 410.3913
70-79	−78.92821	182.5277	−0.43	.665	−436.676 to 278.8195
>80	59.02592	226.9982	0.26	.795	−385.8824 to 503.9342
BMI					
Overweight	207.6795	95.78029	2.17	.030	19.95362 to 395.4054
Obese	258.0722	113.3226	2.28	.023	35.96402 to 480.1804
Morbidly obese	1257.968	623.3053	2.02	.044	36.31178 to 2479.624
Smoking status					
Previous	−57.20011	97.19538	−0.59	.556	−247.6996 to 133.2993
Current	−241.9663	122.4042	−1.98	.048	−481.8741 to −2.058529
Constant	2348.796	305.0676	7.70	.000	1750.875 to 2946.718

Abbreviations: BMI = body mass index; CI = confidence interval; SE = standard error; TCC = transitional cell carcinoma.

**Supplemental Table 12:** Variance–Covariance Matrix Base Case HRQoL Regression Analysis

Variable	EQ-5D Base	Gender	Age, y				BMI			Smoking		ECG Result	Celecoxib	Diabetes	History	Year		Grade				Progression	Progression History	Constant
			50-60	60-70	70-80	>80	Overweight	Obese	Morbidly Obese	Previous	Current					2	3	Unknown	1	2	3			
EQ-5D baseline	0.00165																							
Gender	0.0000	0.0003																						
Age, y																								
50-60	0.0000	−0.0001	0.0011																					
60-70	−0.0001	−0.0001	0.0009	0.0010																				
70-80	0.0000	−0.0001	0.0009	0.0009	0.0011																			
>80	−0.0001	−0.0001	0.0009	0.0009	0.0010	0.0016																		
BMI																								
Overweight	0.0000	0.0000	0.0000	0.0000	0.0000	−0.0001	0.0003																	
Obese	0.0001	0.0000	0.0000	0.0000	0.0000	0.0000	0.0002	0.0003																
Morbidly obese	−0.0001	0.0000	−0.0001	−0.0001	0.0000	0.0000	0.0002	0.0002	0.0042															
Smoking																								
Previous	0.0000	0.0000	−0.0001	−0.0001	0.0000	0.0000	0.0000	0.0000	−0.0001	0.0002														
Current	0.0001	−0.0001	0.0000	0.0000	0.0001	0.0000	0.0000	0.0000	−0.0001	0.0001	0.0006													
ECG result	0.0000	0.0000	0.0000	−0.0001	−0.0001	−0.0002	0.0000	0.0000	0.0000	0.0000	0.0000	0.0003												
Celecoxib	0.0000	0.0000	0.0000	0.0001	0.0000	0.0000	0.0000	0.0000	0.0000	0.0000	−0.0001	0.0000	0.0002											
Diabetes	0.0001	0.0000	0.0000	−0.0001	−0.0001	−0.0001	0.0000	0.0000	−0.0002	0.0000	0.0000	0.0000	0.0000	0.0006										
TCC history	0.0001	0.0000	0.0000	0.0000	0.0000	0.0000	0.0000	0.0000	0.0001	0.0000	0.0000	0.0000	0.0000	0.0000	0.0002									
Year																								
2	0.0000	0.0000	0.0000	0.0000	0.0000	0.0000	0.0000	0.0000	0.0000	0.0000	0.0000	0.0000	0.0000	0.0000	0.0000	0.0001								
3	0.0000	0.0000	0.0000	0.0000	0.0000	0.0000	0.0000	0.0000	0.0000	0.0000	0.0000	0.0000	0.0000	0.0000	0.0000	0.0000	0.0001							
Grade																								
Unknown	0.0000	0.0000	0.0000	0.0000	0.0000	0.0000	0.0000	0.0000	0.0000	0.0000	0.0000	0.0000	0.0000	0.0000	0.0000	0.0000	0.0000	0.0068						
1	0.0000	0.0000	0.0000	0.0000	0.0000	0.0000	0.0000	0.0000	0.0000	0.0000	0.0000	0.0000	0.0000	0.0000	0.0000	0.0000	0.0000	0.0000	0.0031					
2	0.0000	0.0000	0.0000	0.0000	0.0000	0.0000	0.0000	0.0000	0.0000	0.0000	0.0000	0.0000	0.0000	0.0000	0.0000	0.0000	0.0000	0.0000	0.0000	0.0012				
3	0.0000	0.0000	0.0000	0.0000	0.0000	0.0000	0.0000	0.0000	−0.0001	0.0000	0.0000	0.0000	0.0000	0.0000	0.0000	0.0000	0.0000	0.0000	0.0000	0.0000	0.0007			
Progression	0.0000	0.0000	0.0000	0.0000	0.0000	0.0000	0.0000	0.0000	0.0000	0.0000	0.0000	0.0000	0.0000	0.0000	0.0000	0.0000	0.0000	0.0000	0.0000	0.0000	−0.0001	0.0014		
Progression history	0.0000	0.0000	0.0000	0.0000	−0.0001	0.0000	0.0000	0.0000	0.0000	0.0000	0.0000	0.0000	0.0000	0.0000	0.0000	0.0000	0.0000	0.0000	0.0000	0.0000	0.0000	0.0004	0.0027	
Constant	−0.0014	−0.0002	−0.0008	−0.0008	−0.0008	−0.0008	−0.0001	−0.0002	0.0000	0.0000	−0.0001	0.0000	−0.0001	−0.0001	−0.0001	0.0000	0.0000	0.0000	0.0000	0.0000	0.0000	0.0000	0.0000	0.0024

Abbreviations: BMI = body mass index; CI = confidence interval; ECG = electrocardiography; EQ-5D = EuroQoL 5 dimension; HRQoL = health-related quality of life; SE = standard error; TCC = transitional cell carcinoma.

**Supplemental Table 13:** Variance–Covariance Matrix Base Case Cost Regression Analysis

Variable	Tumor Grade				HR	Year		#Year		Progression	Progression History	TCC History	Gender	Diabetes	Celecoxib	Toxicity		#Toxicity		Age, y				BMI			Smoking		Constant
	Unknown	1	2	3		2	3	2	3							Mild	Moderate	Mild	Moderate	50-60	60-70	70-80	>80	Overweight	Obese	Morbidly Obese	Previous	Current	
Tumor grade																													
Unknown	2989584																												
1	3197.897	173025.4																											
2	455.0007	5153.004	148982.9																										
3	4751.888	1440.31	5766.639	687863.1																									
HR	−12,708.1	7947.394	6233.751	−12,978.7	97,028.53																								
Year																													
2	128.5532	2513.226	5118.316	2360.683	45,236.91	63,355.2																							
3	417.7922	6185.773	9399.375	3398.295	46,386.92	47,142.29	54,752.65																						
HR # year 2	12,903.19	−2631.28	−1898.03	13,670.3	−92,607.2	−61,490.2	−44,871.9	118204.4																					
HR # year 3	10,099.37	−5412.12	−5798.32	12,242.9	−94,413.4	−44,807.9	−51,859.6	91,808.03	102383.7																				
Progression	3772.371	67.20135	−12,774.8	−87,906.4	−11,840.8	−1096.95	−1184.22	6543.22	10,696.11	1,960,938																			
Progression history	3597.378	131.9645	1131.308	3651.328	−756.922	429.5359	37.94244	−3169.66	−1512.31	47,298.56	651011.4																		
TCC history	−3973.36	177.3514	−249.955	−618.622	1794.413	201.794	446.6992	−80.3491	−176.578	−1414.4	−2551.17	8372.969																	
Patient gender	−2929.97	−77.8528	500.8079	550.0644	−184.652	924.8961	1028.345	−228.396	−256.491	−934.697	−1450.44	634.9075	10,888.51																
Diabetes	174.1897	458.7002	−786.564	−1579.5	61.53177	93.05056	−59.2129	−127.267	−313.961	1281.531	−1474.4	518.0848	588.3711	21,619.53															
Celecoxib	2048.935	402.4205	−98.0007	920.2471	216.8488	−36.8882	−714.756	81.53749	647.6306	231.6324	515.0038	−10.0378	−759.794	−1101.22	8200.721														
Toxicity																													
Mild	−600.194	429.0679	321.6055	−73.1877	630.1364	4588.025	5054.176	−527.603	−608.46	713.4595	1786.313	298.7808	2207.895	−454.888	3779.813	30,199.89													
Moderate	2383.184	−2263.53	1663.632	2924.52	−1789.75	6931.381	6671.093	103.2672	3430.716	4155.016	1639.651	135.8265	1061.755	−97.2649	3874.048	6976.896	90,355.71												
Celecoxib # mild	438.1648	−1952.97	601.5322	−1065.34	−393.143	−880.25	427.7197	−287.85	−203.163	−1276.02	−266.424	226.7488	−1008.46	427.0523	−7417.36	−29,182.4	−5234.27	58,706.88											
Celecoxib # moderate	−578.645	204.5142	−1252.18	−3234.89	3614.205	−463.231	1200.66	−1325.14	−5724.77	−4619.99	−150.712	−435.541	−60.541	550.1123	−7471.91	−5457.64	−88,242.4	9219.409	150368.5										
Age, y																													
50-60	−461.326	−1297.4	−2167.35	−219.887	877.5002	−383.189	−780.626	−62.6462	536.8991	1446.35	−632.307	54.15569	−388.29	−844.322	1911.414	−315.633	−16.8047	−1357.87	−284.003	37,575.4									
60-70	−760.247	−565.579	−1189.19	−1265.14	1569.057	−532.382	−836.337	80.84493	565.3626	−535.485	−3198.85	294.7149	−1511.36	−2402.45	2058.851	−920.176	−519.123	−1076.13	−61.9788	27,048.9	31,468.3								
70-80	−3417.03	−643.19	−422.365	−493.563	1144.489	−680.322	−1062.53	32.89036	515.9473	−632.644	−2133.45	−233.831	−1363.8	−2793.31	1804.646	−1484.46	−1129.18	375.9521	−995.23	26,940.94	27,342.93	33,316.37							
>80	−2210.66	−1113.08	−169.061	−1806.11	892.5153	−297.992	−451.897	16.11853	386.4642	1645.557	−342.335	140.223	−925.094	−1278.83	2066.425	−156.349	460.59	−827.155	−2429.76	27,010.24	27,247.6	27,388.06	51,528.18						
BMI																													
Overweight	3529.466	573.0519	−339.4	−552.401	250.3219	24.09107	739.0862	109.069	−443.283	491.4164	290.0057	−150.674	−73.1737	−792.842	−161.863	724.8933	−623.956	−1356.18	1204.683	−556.359	−313.425	38.44737	−1558.25	9173.863					
Obese	3521.786	400.006	−595.534	−726.146	−612.32	−32.3199	604.3832	90.40263	−260.271	−246.015	1816.794	−373.677	−373.834	−3086.28	−306.06	117.209	−1073.1	28.07319	1280.047	−2138.66	−816.066	193.7971	−609.137	5204.197	12,842.01				
Morbidly obese	−118.211	−9986.43	−9469.14	−2409.75	−2017.09	40.76192	1044.331	456.362	986.1302	3133.209	1577.672	33.32034	4273.654	−6030.72	−2071.96	2339.3	19.31831	−1655.31	1015.499	4159.043	3032.126	6170.16	5360.11	5745.526	6500.394	388509.5			
Smoking																													
Previous	3051.792	−192.533	−525.105	735.3619	147.8917	−410.739	−357.737	167.0641	−65.6603	−492.734	1603.186	−316.844	−1847.56	−829.911	91.54407	−1454.3	−945.744	1053.433	−61.0395	−905.897	−1423.65	−677.65	−1246.05	−968.874	-696.796	−4306.3	9446.943		
Current	3021.291	1286.492	−351.976	619.1337	1659.942	−449.125	−294.024	231.7881	−115.202	−93.4865	785.4893	216.0982	−2526.05	−1053.26	−581.953	−978.082	−1439.44	1179.949	787.8671	1593.945	1184.458	2831.625	2421.957	409.944	639.0463	−575.662	5894.281	14,982.78	
Constant	−650.554	−8561.44	−9019.26	−3331.26	−50,134.5	−48,152.1	−49,973.6	45,313.96	46,188.36	2439.925	2001.973	−4320.44	−6659.01	1944.855	−5262.25	−9175.75	−10,217.5	5459.517	3663.643	−26,755.5	−26,623.3	−26,969.7	−26,842.4	−4592.3	−3397.78	−9238.54	−2227.81	−6309.84	93,066.23

Abbreviations: BMI = body mass index; CI = confidence interval; ECG = electrocardiography; HR = high risk; HRQoL = health-related quality of life; SE = standard error; TCC = transitional cell carcinoma.
